# Genomic characterization of selected influenza A-positive specimens and an A(H1N1)pdm09/SARS-CoV-2 co-infection in the Republic of Moldova

**DOI:** 10.3389/fpubh.2026.1913939

**Published:** 2026-09-07

**Authors:** Svetlana Colac, Valeria Ceban, Livia Țapu, Veronica Burlac, Nicolae Jelamschi, Olga Burduniuc

**Affiliations:** 1 National Agency for Public Health, Chisinau, Moldova; 2 Nicolae Testemitanu State University of Medicine and Pharmacy, Chisinau, Moldova

**Keywords:** co-infection, genomic surveillance, influenza A, respiratory viruses, SARS-CoV-2

## Abstract

**Background:**

Genomic sequencing can complement routine influenza surveillance by enabling clade-level characterization of selected specimens and the genomic characterization of mixed viral specimens. This descriptive laboratory-based study characterized influenza A-positive respiratory specimens collected through routine surveillance in the Republic of Moldova and selected for sequencing.

**Methods:**

Between October 2025 and February 2026, 1,081 respiratory specimens were screened by multiplex real-time reverse transcription polymerase chain reaction for influenza A, influenza B, and SARS-CoV-2. Of 291 influenza A-positive specimens, 174 had cycle-threshold values ≤27, and 38 were selected for hybrid-capture sequencing based on repeat cycle-threshold values and technical suitability. Consensus sequences were analyzed with Nextclade, and the SARS-CoV-2 component of one mixed specimen was additionally assessed using Pangolin.

**Results:**

All 38 selected specimens yielded analyzable influenza A hemagglutinin sequences, with hemagglutinin-segment coverage ranging from 87.6% to 100%. The dataset comprised 32 A(H3N2) specimens, five A(H1N1)pdm09 specimens, and one mixed A(H1N1)pdm09/SARS-CoV-2 specimen. All A(H3N2) hemagglutinin sequences were assigned to clade K, subclade 2a.3a.1. The six A(H1N1)pdm09 hemagglutinin components were assigned to clade D.3.1 (*n* = 2) or D.3.1.1 (*n* = 4). The SARS-CoV-2 consensus was assigned to Nextclade clade 25I; poor overall sequence quality limited confidence in fine-scale lineage assignment and precluded robust interpretation of private mutations.

**Conclusion:**

The study provides a descriptive genomic characterization of a laboratory-selected subset and illustrates a workflow linking molecular testing to sequencing. The findings should not be interpreted as estimates of national clade prevalence, geographical distribution, or A(H1N1)pdm09/SARS-CoV-2 co-infection frequency.

## 1 Introduction

Respiratory viruses remain a major public health threat because of their seasonal circulation, genetic variability, recurrent emergence, and capacity to drive overlapping epidemics. Seasonal influenza alone causes approximately 1 billion infections worldwide each year, including 3–5 million severe cases and 290,000–650,000 respiratory deaths (1). Since the emergence of severe acute respiratory syndrome coronavirus 2 (SARS-CoV-2), surveillance of acute respiratory illness has increasingly required an integrated approach, as influenza viruses, SARS-CoV-2, respiratory syncytial virus (RSV), and other respiratory pathogens may co-circulate and cause clinically similar disease manifestations (2 – 4).

The World Health Organization (WHO) has strengthened this approach through its operational guidance on the integrated sentinel surveillance of influenza and other respiratory viruses with epidemic and pandemic potential within the Global Influenza Surveillance and Response System (GISRS). Under this model, integrated surveillance covers the full pathway from screening, sample collection and laboratory testing to sequencing, data sharing and analysis (2). The WHO Global Genomic Surveillance Strategy for Pathogens with Pandemic and Epidemic Potential, 2022–2032, further emphasizes that genomic sequencing should be embedded within broader surveillance and laboratory systems, with sustained capacity for specimen collection, diagnosis, analysis, interpretation and data sharing (3). In Europe, the European Center for Disease Prevention and Control (ECDC) recommends integrated reporting for influenza, coronavirus disease 2019 (COVID-19), and other respiratory viruses, including RSV, across European Union/European Economic Area (EU/EEA) countries (4).

The Republic of Moldova has an established surveillance system for influenza, acute respiratory infections, and severe acute respiratory infections. A national analysis covering the 2014/15–2022/23 seasons documented the circulation of A(H1N1)pdm09, A(H3N2), and influenza B viruses, highlighting the importance of surveillance for influenza-like illness (ILI), acute upper respiratory tract infection (AURTI), and severe acute respiratory infection (SARI) in real-time epidemic monitoring and public health decision-making (5).

Building genomic sequencing into this surveillance framework enables clade-level characterization, comparison of national findings with regional trends, and detection of co-circulation or co-infection events with epidemiological relevance.

National genomic surveillance for SARS-CoV-2 has been documented in several local scientific works. A 2023 publication reported the analysis of 416 samples collected in 2022 using fragment sequencing on the Ion Torrent Genexus platform, with Pangolin lineage assignment and clade classification according to the Global Initiative on Sharing All Influenza Data (GISAID) nomenclature. The study described the replacement of Delta by Omicron and the subsequent circulation of BA.1, BA.2, BA.4/BA.5 and their descendant lineages, including BQ.1, BN.1, CC.1 and XBB.1.5 (6, 7). Other studies underlined that the ongoing evolution of the SARS-CoV-2 genome, particularly in the spike protein and receptor-binding domain, supports the need for genetic monitoring to inform diagnostics, assessment of immune escape and public health response (8, 9).

In 2025, a national study further expanded this experience by sequencing 71 SARS-CoV-2 samples collected between December 2023 and August 2024. The analysis identified 10 clades and 18 genetic lineages, with JN.1 predominating in the first part of 2024 and JN.1.11.1 and KP.2 becoming more prominent between June and August (10).

Recent studies have further examined the public health implications of emerging Omicron lineages, the use of wastewater-based surveillance for SARS-CoV-2 monitoring, and the application of metagenomic approaches to the detection of clinically relevant microorganisms and antimicrobial resistance. Collectively, this body of work reflects the gradual consolidation of a national genomic sequencing infrastructure with potential application in integrated pathogen surveillance (11–14). Earlier reports published in 2022 on whole-genome sequencing and phylogenetic analysis of SARS-CoV-2 isolates provided the operational foundation for the subsequent integration of next-generation sequencing (NGS) into national surveillance activities (15, 16).

In influenza surveillance, genomic characterization of the hemagglutinin (HA) and neuraminidase (NA) gene segments complements reverse transcription polymerase chain reaction (RT-PCR)-based diagnosis by enabling clade assignment and the identification of substitutions with antigenic, epidemiological, or potential functional significance. Sequence sharing through GISAID has become essential for global comparison of influenza viruses and for the contribution of national laboratories to international surveillance networks (17). Bioinformatic platforms such as Nextclade support standardized alignment, mutation calling, quality control (QC), and clade assignment, thereby improving the reproducibility of genomic analyses (18). For SARS-CoV-2, phylogenetic classification is complemented by the Pango lineage nomenclature, implemented through the Pangolin software, which was developed to describe the expanding diversity of SARS-CoV-2 lineages and to support genomic epidemiology (19).

The recent European context has been shaped by the increasing relevance of influenza A(H3N2) subclade K, which has shown genetic divergence from the A(H3N2) strain included in the 2025/26 northern hemisphere vaccine formulation and has become prominent in several European countries (20–22). WHO subsequently updated its recommendations for the composition of influenza vaccines for the 2026/27 northern hemisphere season on the basis of global surveillance and virus characterization data (23). In November 2025, ECDC reported increasing circulation of A(H3N2) subclade K in the EU/EEA, noting uncertainty regarding its clinical impact while recommending continued vaccination and routine prevention measures (20). Early European vaccine-effectiveness data for the 2025/26 season suggested moderate protection against influenza A overall, despite the predominance of A(H3N2) subclade K (21). A recent narrative review further described subclade K as a rapidly evolving antigenic variant of relevance to public health in Europe (22).

Influenza/SARS-CoV-2 co-infection is clinically and epidemiologically relevant, as detection of one respiratory virus does not exclude the presence of another. Cases of SARS-CoV-2/influenza A co-infection were reported early in the pandemic (24). Evidence from a national observational study in England and a multicentre clinical cohort of hospitalized COVID-19 patients in the United Kingdom showed that concurrent infection with SARS-CoV-2 and influenza virus was associated with an increased risk of severe outcomes, including mechanical ventilation, intensive care admission, or death, compared with SARS-CoV-2 mono-infection (25, 26). However, systematic reviews and meta-analyses have shown substantial variation in reported frequency and in the association with disease severity, depending on the study population, period of observation, testing intensity, and concurrent circulation of respiratory viruses (27–29). These findings should therefore not be extrapolated directly to individual cases.

The aim of this descriptive laboratory-based study was to genomically characterize 38 influenza A-positive respiratory specimens collected through routine surveillance in the Republic of Moldova and subsequently selected in the laboratory for sequencing; to describe their geographical origins and available anonymized epidemiological metadata; to assign A(H1N1)pdm09 and A(H3N2) HA clades; and to report one mixed specimen containing A(H1N1)pdm09 and SARS-CoV-2.

The study was not designed to estimate national clade prevalence or predominance, geographical distribution, or the frequency of influenza A/SARS-CoV-2 co-infection.

## 2 Materials and methods

### 2.1 Study design

This descriptive laboratory-based surveillance study was conducted using nasopharyngeal and oropharyngeal swab samples collected between October 2025 and February 2026 through the routine respiratory virus surveillance system in the Republic of Moldova. Samples were obtained as part of national surveillance for influenza-like illness (ILI), acute upper respiratory tract infection (AURTI) and severe acute respiratory infection (SARI).

The genomic analysis was restricted to influenza A and to the SARS-CoV-2 component detected in one mixed specimen. Initial systematic molecular testing included influenza A, influenza B, and SARS-CoV-2. Respiratory syncytial virus, parainfluenza viruses, adenoviruses, and other respiratory viruses were not systematically tested. The study could not assess co-detections involving respiratory viruses other than influenza viruses and SARS-CoV-2.

### 2.2 Sampling and surveillance framework

The sentinel surveillance catchment denominator comprised persons registered with participating family physicians and was stratified by age. These population denominators describe the surveillance catchment and were not used to estimate clade prevalence, geographical distribution, or co-infection frequency in the laboratory-selected sequencing subset.

Virological surveillance of seasonal influenza, AURTI, SARI, and COVID-19 is carried out throughout the year, within the framework of the sentinel-type epidemiological surveillance system, in 9 administrative territories - sentinel points: Chisinau, Balti, Causeni, Cahul, Comrat, Edinet, Orhei, Soroca and Ungheni.

The population-level denominators are made up of the number of the population by age that is recorded in the lists of family doctors. ILI Denominators are following: 0–4 years – 381,056, 5–14 years – 21,678, 15–64 years – 47,252 and > 65 years – 261,816. The total population covered by the sentinel surveillance system was 711,802.

Biological sample collection is carried out by designated medical and sanitary institutions, on the days and within the limit of the weekly number of samples established for each sentinel point. On the designated days, the sample is collected from the first patient who presents for medical assistance and meets the case definition for influenza/ILI, AURTI, SARI or COVID-19. Severe acute respiratory infection was defined as an acute respiratory infection with a history of fever or measured temperature of at least 38 °C and cough, with symptom onset within the preceding 10 days and requiring hospitalization, in accordance with Ministry of Health Order No. 897 of 8 October 2025 (30).

The time interval from sample collection to confirmation of the result did not exceed 72 h, in accordance with the recommendations of the guide Implementing the Integrated Sentinel Surveillance of Influenza and Other Respiratory Viruses of Epidemic and Pandemic Potential by the Global Influenza Surveillance and Response System (pp. 12–13) and with the provisions of the Order of the Ministry of Health no. 897 of 08.10.2025 On epidemiological surveillance of acute viral respiratory infections (2, 30).

Processing of samples and carrying out investigations using molecular biology techniques, from the moment of their receipt in the laboratory until the issuance of the result, is carried out within a maximum of 24 h, all samples received being investigated and reported on the same day.

Samples are collected within the first 4 days of illness in cases of influenza, AURTI and COVID-19 and up to 10 days from onset in cases of SARI. For each sample and patient, the accompanying bulletin provided for in Annex No. 4 from the Order of the Ministry of Health No. 897 of 08.10.2025 on the epidemiological surveillance of acute viral respiratory infections (30).

Between October 2025 and February 2026 there was received a total number of 1,081 ILI, AURTI and SARI samples, from which 928 samples were received form sentinel points. This entire number of samples was also investigated for influenza and SARS-CoV-2. During the analyzed period, 291 positive samples for influenza A virus (sentinel and non-sentinel) were confirmed, of which: Influenza A(H1N1)pdm09–41 positive samples; influenza A(H3N2)—186 positive samples; non-subtyped influenza A—64 positive samples. Additionally, for SARS-CoV-2 – 8 positive samples. One of the A(H1N1)pdm09-positive specimens was also positive for SARS-CoV-2, representing an A(H1N1)pdm09/SARS-CoV-2 co-infection. Therefore, the influenza A-positive and SARS-CoV-2-positive counts were not mutually exclusive.

Specimens were submitted by healthcare institutions and district laboratories to the National Reference Laboratory for Viral Respiratory and Enteric Infections of the National Agency for Public Health for laboratory confirmation. All specimens were initially screened by multiplex real-time RT-PCR using the TaqPath™ COVID-19, FluA, FluB Combo Kit (Applied Biosystems/Thermo Fisher Scientific), which includes targets for influenza A, influenza B, and SARS-CoV-2. Influenza A-positive specimens considered for sequencing were retested before transfer to the genomic sequencing laboratory using influenza real-time RT-PCR primer/probe reagents developed by the United States Centers for Disease Control and Prevention and supplied through the International Reagent Resource. The repeat assays targeted universal influenza A, the A(H1N1)pdm09-specific H1 target, and the A(H3N2)-specific H3 target and were performed using TaqPath™ 1-Step Multiplex Master Mix. Target-specific fluorescence was measured in the 6-carboxyfluorescein channel. Retesting was used to confirm influenza A type or subtype and to obtain the cycle-threshold (Ct) values used for sequencing eligibility and enrichment-pool allocation. The Illumina Viral Surveillance Panel v2 was used for library preparation and hybrid-capture sequencing and was not used for initial diagnostic detection or RT-PCR confirmation.

Ministry of Health Order No. 897 of 8 October 2025 establishes the general national framework for prioritizing influenza virus and SARS-CoV-2 specimens for genomic sequencing. The framework includes epidemiological and laboratory criteria, including geographical and temporal distribution, severe cases, unusual epidemiological links, outbreaks in high-risk groups, information concerning vaccine or antiviral-treatment effectiveness, and Ct values below 25–27, depending on the amplification assay. The Order also specifies that at least 5–10% of confirmed positive specimens should be sequenced (30).

For the present study, the broader national epidemiological criteria were treated as part of the surveillance framework and were not systematically applied as individual study-level inclusion variables. Operational inclusion in the sequencing subset was based primarily on influenza A positivity confirmed by repeat RT-PCR, Ct values compatible with sequencing, sufficient residual specimen volume and integrity, appropriate transport and storage conditions, availability of minimum anonymized metadata, and compatibility with the Ct-based enrichment and sequencing-run configuration.

Of the 291 influenza A-positive specimens, 174 had Ct values ≤27 and met the basic laboratory eligibility criterion. From this Ct-eligible group, 38 specimens were included based on repeat Ct values, compatibility with the planned enrichment configuration, and the technical capacity of the two sequencing runs. The 38 specimens represented 13.1% of all influenza A-positive specimens identified during the study period. They constituted a non-probability, laboratory-selected subset and were not considered statistically representative.

### 2.3 Metadata and confidentiality

Available metadata included the laboratory sample code, locality or district of origin, submitting institution, clinical or surveillance category, date of symptom onset, date of sample collection, date of testing, age group, reported vaccination status, and RT-PCR Ct values. For descriptive analysis, age was categorized as <1 year, 1–4 years, 5–17 years, 18–49 years, 50–64 years, and ≥65 years. Specimens for which age could not be reliably derived were classified as having insufficient age data. The diagnostic or surveillance category represented the presumptive clinical or surveillance classification recorded at specimen submission and was analyzed separately from the laboratory detection result.

Direct identifiers, including names, personal identification numbers, and addresses, were removed before analysis for the manuscript. Tables report only aggregate data or laboratory sample codes, with no information that could allow individual identification.

### 2.4 Sample collection and ribonucleic acid extraction

The collected biological material was the combined nasopharyngeal exudate, consisting of a nasal swab and a pharyngeal swab. Both swabs are placed in a single sterile tube containing viral transport medium, respecting the principle of “1 tube/1 patient.”

Viral ribonucleic acid (RNA) from SARS-CoV-2 and influenza A-positive samples was extracted from 140 μL of clinical specimen using the QIAamp Viral RNA Mini Kit (QIAGEN), according to the manufacturer’s instructions. Extracted RNA was eluted in 60 μL of Buffer AVE and stored at −70 °C until sequencing.

### 2.5 Library preparation and sequencing

Deoxyribonucleic acid (DNA) libraries were prepared using the Illumina Viral Surveillance Panel v2, according to the manufacturer’s instructions. This panel enables the detection and whole-genome sequencing of more than 200 viruses. It is based on a hybrid-capture target enrichment workflow, which allows sequencing across different sample types without requiring the high read depth typically needed for shotgun metagenomic sequencing. Library preparation with the Illumina Viral Surveillance Panel v2 involved two main steps: pre-enrichment and enrichment. During pre-enrichment, large numbers of untargeted libraries are generated and subsequently enriched using Viral Surveillance Panel v2 probes through a hybrid-capture approach. Magnetic bead-based capture enrichment provides a rapid, automation-compatible workflow that can be completed in approximately 2 days with minimal hands-on time. The protocol accepts a total nucleic acid input ranging from 10 ng to 100 ng. Specimens with comparable repeat Ct values were grouped into 3-plex enrichment pools, in accordance with the manufacturer’s recommendations, to minimize potential imbalance associated with differences in relative viral input among pooled specimens.

Hybrid-capture sequencing. Prepared DNA libraries were sequenced on an Illumina MiSeq Dx next-generation sequencing platform using the MiSeq Reagent Kit v3, 600 cycles. Libraries were normalized to a starting concentration of 4 nM and loaded at a final concentration of 10–12 pM. Sequencing was performed in paired-end mode, generating 300 × 150 bp reads. The 38 specimens were sequenced in two independent MiSeq runs: the first run included 22 specimens and the second included 16 specimens. The allocation was based on the established multiplexing configuration of the laboratory workflow, with a maximum of 24 influenza specimens per run, and was intended to maintain the recommended library-preparation and loading parameters and to ensure adequate per-specimen sequencing performance and coverage.

Base calling, demultiplexing, and generation of sequence-read files containing nucleotide sequences and quality scores (FASTQ files) were carried out on the MiSeq Dx instrument after completion of the sequencing run. Sequencing output, including FASTQ files, was automatically uploaded to Illumina BaseSpace™ Sequence Hub for run and project data storage.

### 2.6 Bioinformatic analysis and phylogenetic profiling

Bioinformatic analysis of sequencing data was performed using open-access international databases and validated analysis platforms. Host-read filtering, alignment to reference genomes, taxonomic detection, and estimation of relative abundance were carried out using the *Dynamic Read Analysis for GENomics (DRAGEN™) Microbial Enrichment Plus application* (v1.1.1) available through *BaseSpace™ Sequence Hub.* Assembled consensus sequences in text-based FASTA nucleotide-sequence format were further analyzed using *Nextclade* (v3.21.2) and, for the SARS-CoV-2 sequence, Pangolin. These tools were used for clade and subclade assignment according to GISAID/Nextstrain nomenclature and for SARS-CoV-2 lineage classification. The exact Pangolin software and lineage-assignment data versions were not retained in the available output.

The influenza A(H1N1) sequences were analyzed on Nextclade with “Influenza A(H1N1) pdm HA (nextstrain/flu/h1n1pdm/ha/MW626062)” dataset, “A/Wisconsin/588/2019 (MW626062)” reference strain. Influenza A(H3N2) samples were analyzed with “Influenza A H3N2 HA (nextstrain/flu/h3n2/ha/EPI1857216)” dataset, “A/Darwin/6/2021 (EPI1857216)” reference strain and SARS-CoV-2 sample was analyzed with “SARS-CoV-2 (BA2.86; nextstrain/sars-cov-2/BA.2.86)” dataset, “Wuhan-Hu-1 with BA.2.86 SNPs” reference strain. In this dataset name, SNPs refers to single-nucleotide polymorphisms.

Although the Illumina Viral Surveillance Panel v2 supports hybrid-capture sequencing across influenza genome segments, the downstream influenza analysis in this study was prespecified to focus on the hemagglutinin segment. The primary analytical objectives were HA-based clade assignment, characterization of genetic variation within HA, and evaluation of HA-based phylogenetic relationships. Accordingly, subtype-specific HA datasets were used in Nextclade for A(H1N1)pdm09 and A(H3N2). The neuraminidase segment and the remaining influenza genome segments were not included in the final downstream analysis. Therefore, substitutions associated with resistance to oseltamivir or other neuraminidase inhibitors were not assessed, and no conclusions regarding antiviral susceptibility were made.

The following minimum sequence-retention criteria were applied: genome coverage: ≥ 80%; Ambiguous Bases: < 10%; average nucleotide identity (ANI): ≥95%; Nextclade quality-control (QC) status: good (overall score <29) or mediocre (overall score 29–100). The first sequencing run was analyzed on 16 January 2026, and the second sequencing run was analyzed on 7 March 2026.

Phylogenetic trees for Influenza A and SARS-CoV-2 were generated and visualized in Nextclade, which places query sequences within reference phylogenies based on alignment to globally representative reference strains.

### 2.7 Statistical analysis

The analysis was descriptive. Categorical variables were summarized as absolute numbers and proportions. No tests of statistical significance were performed, as the study was based on bioinformatic analysis of the sequences obtained, and the sample was small, laboratory-selected, and not linked to population-level epidemiological denominators.

## 3 Results

### 3.1 Distribution of samples and viral components

Among the 38 sequenced respiratory specimens, 32 (84.2%) were classified as A(H3N2), 5 (13.2%) as A(H1N1)pdm09, and one specimen (2.6%) as influenza A/SARS-CoV-2 co-infection, representing A(H1N1)pdm09/SARS-CoV-2 co-infection. Because the influenza A/SARS-CoV-2 co-infection specimen contained both an A(H1N1)pdm09 component and a SARS-CoV-2 component, genomic results are reported separately for each viral component. Overall, the dataset comprised 32 A(H3N2) components analyzed for the HA segment, 6 A(H1N1)pdm09 components analyzed for the HA segment, and one SARS-CoV-2 component analyzed at consensus-genome level (Table 1).

**Table 1:** Distribution of biological specimens and genomically analyzed viral components.

Reporting unit	Category	Nr	% of 38 specimens	Genomic interpretation
Biological specimens	A(H3N2) mono-infection	32	84,2	Clade K; subclade 2a.3a.1
Biological specimens	A(H1N1)pdm09, mono-infection	5	13,2	D.3.1 or D.3.1.1
Biological specimens	A(H1N1)pdm09 + SARS-CoV-2 co-infection	1	2,6	A(H1N1)pdm09 D.3.1.1 + SARS-CoV-2 Omicron 25I/SJ.1
Biological specimens	Total specimens	38	100,0	—
Viral components included in genomic analysis	A(H3N2), HA segment	32	—	32/32 clade K
Viral components included in genomic analysis	A(H1N1)pdm09, HA segment	6	—	2 D.3.1; 4 D.3.1.1, including the H1N1 component of the Influenza A/SARS-CoV-2 co-infection specimen
Viral components included in genomic analysis	SARS-CoV-2, consensus genome	1	—	Omicron 25I/SJ.1; poor overall QC

HA, hemagglutinin; QC, quality control; SARS-CoV-2, severe acute respiratory syndrome coronavirus 2. For influenza viruses, clade assignment was based on the HA segment. For SARS-CoV-2, assignment was based on the available consensus genome. Percentages are reported only for biological specimens; viral components are reported as the number of sequences or consensus genomes analyzed.

### 3.2 Sample origin and metadata characteristics

The specimens originated from 13 regions, municipalities, or districts. The largest proportion was from Chișinău (*n* = 17; 44.7%), followed by Bălți and Orhei, with 4 specimens each (10.5%), and Edineț (*n* = 3; 7.9%). The influenza A/SARS-CoV-2 co-infection specimen was collected in Chișinău. The distribution by locality/district of origin is shown in Table 2.

**Table 2:** Distribution of specimens by geographical origin and detected virus type.

Locality / district / municipality	A(H1N1)	A(H3N2)	Influenza A/SARS-CoV-2 co-infection	Total	% of total
Chișinău	3	13	1	17	44,7
Bălți	0	4	0	4	10,5
Comrat	0	1	0	1	2,6
Criuleni	1	0	0	1	2,6
Căușeni	0	1	0	1	2,6
Drochia	0	1	0	1	2,6
Edineț	0	3	0	3	7,9
Fălești	0	1	0	1	2,6
Ialoveni	0	1	0	1	2,6
Orhei	0	4	0	4	10,5
Rîșcani	1	1	0	2	5,3
Soroca	0	1	0	1	2,6
Strășeni	0	1	0	1	2,6
Total	5	32	1	38	100,0

The Influenza A/SARS-CoV-2 co-infection case is reported as a separate biological specimen and was not included among A(H1N1)pdm09 mono-infections.

Specimens were distributed across the predefined numerical age categories shown in Table 3. The 5–17-year age group accounted for 10 specimens (26.3%) and included the mixed A(H1N1)pdm09/SARS-CoV-2 specimen.

**Table 3:** Epidemiological and clinical characteristics of the included specimens.

Variable	Category	A(H1N1)	A(H3N2)	Influenza A/SARS-CoV-2 co-infection	Total n (%)
Age	<1 year	0	3	0	3 (7,9)
	1–4 years	1	4	0	5 (13,2)
	5–17 years	0	9	1	10 (26,3)
	18–49 years	0	6	0	6 (15,8)
	50–64 years	2	3	0	5 (13,2)
	≥ 65 years	0	4	0	4 (10,5)
	Insufficient data	2	3	0	5 (13,2)
Diagnosis/surveillance	Influenza	4	21	1	26 (68,4)
	AURTI	1	9	0	10 (26,3)
	SARI	0	1	0	1 (2,6)
	COVID-19	0	1	0	1 (2,6)
Vaccination status	Vaccinated	0	4	0	4 (10,5)
	Unvaccinated	3	23	1	27 (71,1)
	Not reported / unknown	2	5	0	7 (18,4)

AURTI, acute upper respiratory tract infection; COVID-19, coronavirus disease 2019; SARI, severe acute respiratory infection; SARS-CoV-2, severe acute respiratory syndrome coronavirus 2. “Insufficient data” indicates that the date of birth or the relevant reference date was missing or could not be validated.

Vaccination status was reported as vaccinated for 4 specimens (10.5%), unvaccinated for 27 specimens (71.1%), and undeclared or unknown for 7 specimens (18.4%). Of the 38 specimens analyzed, 26 were submitted under the influenza diagnostic/surveillance category, 10 under the acute upper respiratory tract infection category, one under the severe acute respiratory infection category, and one under the COVID-19 diagnostic/surveillance category. The specimen recorded under the COVID-19 category was specimen 40,000,448, in which A(H1N1)pdm09 and SARS-CoV-2 were simultaneously detected.

### 3.3 RT-PCR Ct values

Ct values varied according to the molecular target and the specimen analyzed. Among A(H1N1)pdm09 mono-infection specimens, the median Ct value was 23.37 for the influenza A target and 23.15 for the primary H1 target. For A(H3N2), the median Ct value was 22.00 for the influenza A target and 23.18 for the repeated H3 target. The influenza A/SARS-CoV-2 co-infection specimen showed a Ct value of 18.65 for the primary H1 target and 26.47 for SARS-CoV-2 (Table 4).

**Table 4:** Cycle-threshold values obtained by real-time reverse transcription polymerase chain reaction for sequenced respiratory specimens.

Category	No. of specimens	Ct influenza A, median (min–max)	Ct H1 primary	Ct H1 repeated	Ct H3 primary	Ct H3 repeated	Ct SARS-CoV-2
A(H1N1)pdm09, mono-infection	5	23,37 (21,00–26,20); *n* = 5	23,15 (18,85–26,01); *n* = 5	26,24 (25,05–27,08); *n* = 5	Not applicable	Not applicable	Not applicable
A(H3N2), mono-infection	32	22,00 (14,28–25,52); *n* = 27	Not applicable	Not applicable	22,90 (16,69–28,75); *n* = 23	23,18 (14,29–30,39); *n* = 32	Not applicable
A(H1N1)pdm09 + SARS-CoV-2	1	25,24	18,65	25,91	Not applicable	Not applicable	26,47

Ct, cycle threshold; H1, A(H1N1)pdm09-specific target; H3, A(H3N2)-specific target. For categories represented by a single specimen, individual cycle-threshold values are reported.

### 3.4 Sequence quality and HA-based influenza characterization

All samples were successfully sequenced across two sequencing runs, resulting in a sample-level sequencing failure rate of 0%. The cluster densities was 1,520 K/mm^2^ in the first run and 1,164 K/mm^2^ in the second run. The proportions of clusters passing the quality filter were 83.1 and 90.55%, respectively, while the percentages of bases with a Phred quality score of Phred quality scores of at least 30 (Q30) or higher were 88.34 and 91.92%, respectively. Hybrid-capture sequencing generated data across influenza genome segments; however, downstream influenza quality assessment, clade assignment, mutation reporting, and phylogenetic interpretation in the present study were restricted to the HA segment.

The sequencing results showed full agreement with those provided by RT-PCR. The total number of Raw Reads per sample ranged between 182,742 and 4,780,070, with a proportion of Unique Reads ranging from 43.92 to 98.61%. The number of Aligned Reads to the reference genome ranged from 958 to 1,585,499, yielding an Average Nucleotide Identity (ANI) of 94.70 99.12%. The Median Sequencing Depth recorded values between 6x and 11,599x. Genome coverage for all sequence is 84.39–100%, and the HA-segment coverage is from 87.6 to 100%. The proportion of ambiguous nucleotides is 1.33–6.61%. The number of total Missings for the HA segment is 0 for those samples that have HA segment coverage 100% and from 1 to 213 that have HA segment coverage 87.6–99.9%.

Next,clade analysis of the A(H1N1)pdm09 sequences showed good Overall score, with few or no deletions, insertions, frameshifts, missing regions, or unknown amino acids, supporting reliable molecular and phylogenetic interpretation of the HA segment (Table 5). For A(H3N2), most sequences showed good overall score and coverage compatible with robust assignment to clade K. One isolate had approximately 87% coverage and a higher number of uncovered positions or unknown amino acids, which limited full interpretation of its mutational profile but did not prevent clade K assignment.

**Table 5:** Genomic characteristics of the viral components analyzed.

Virus	Clade / subclade	No. analyzed	Genetic lineage	No. mutations	Amino acid substitutions	Insertion	Deletions	Main substitutions/remarks
A(H1N1)pdm09	D.3.1	2 HA component	6B.1A.5a.2a.1 / 5a.2a.1	46–48	18–19	1–2	0–2	HA1: T120A; HA1: T216A; HA2: I45V; HA2: I133T; HA2: V193A. One isolate: HA1: D222N.
A(H1N1)pdm09	D.3.1.1	4 HA components and influenza A/SARS-CoV-2 co-infection	6B.1A.5a.2a.1 / 5a.2a.1	53–54	22–27	0–9	0–9	HA1: R113K; HA1: T120A; HA1: A139D; HA1: T216A; HA1: E283K; HA1: K302E; HA2: I45V; HA2: I133T; HA2: V193A. One isolate: non-Influenza A/SARS-CoV-2 co-infection: HA1: K169R.
A(H3N2)	K	32 HA components	3C.2a1b.2a.2a.3a.1 / 2a.3a.1	37–46	14–39	4–39	0–17	HA1, HA1, HA1, HA1, HA1, and HA2 were detected, with internal diversification into three clusters.
SARS-CoV-2	25I/Omicron; Pango SJ.1	1 viral component	BA.3.2.2.1.1.2.1	165	23	18	87	ORF7a, ORF8, and S were reported as consensus artifacts.

HA, hemagglutinin; HA1 and HA2, hemagglutinin subunits 1 and 2, respectively; ORF, open reading frame; S, spike protein; SARS-CoV-2, severe acute respiratory syndrome coronavirus 2; WHO, World Health Organization. For Influenza, lineages and clades are reported according to Nextclade/Nextstrain/WHO nomenclature. For SARS-CoV-2, Pango/Pangolin classification was applied only to the SARS-CoV-2 component. The final Pango assignment for the SARS-CoV-2 component should be confirmed after the final Pangolin export, as the working files contain closely related terminal sublineage designations.

The SARS-CoV-2 component showed a good coverage (97.08%), that was sufficient for phylogenetic analysis and clade assignment. However, Overall score was Bad (>100), with a high number of mutations, deletions, frameshifts, and uncovered positions (838 total Missings). Therefore, private mutations in this component should be reported as consensus artifacts. (Table 5).

### 3.5 Characterization of A(H1N1)pdm09

All six A(H1N1)pdm09 sequences analyzed for the HA segment belonged to lineage 6B.1A.5a.2a.1. Two sequences were assigned to clade D.3.1 and four to clade D.3.1.1. In the influenza A/SARS-CoV-2 co-infection specimen, the influenza A(H1N1)pdm09 component was assigned to clade D.3.1.1 within evolutionary lineage 5a.2a.1, whereas the SARS-CoV-2 component was classified as Omicron BA.3.2, Nextstrain clade 25I, Pango/Nextclade sublineage SJ.1, and full Pango lineage BA.3.2.2.1.1.2. In the mutation notation used below, HA1 and HA2 refer to hemagglutinin subunits 1 and 2, respectively. All A(H1N1)pdm09 components carried the HA1: T120A substitution, together with the shared substitutions HA1: T216A, HA2: I45V, HA2: I133T, and HA2: V193A. The D.3.1.1 components also carried the additional HA1 substitutions HA1: R113K, HA1: A139D, HA1: E283K, and HA1: K302E. Compared with the reference sequence, influenza A(H1N1)pdm09 strains assigned to clade D.3.1 showed 46–48 mutations, including 18–19 amino acid substitutions, with few insertions, 1–2, and deletions, 0–2. Strains belonging to clade D.3.1.1 showed a higher number of mutations, 53–54, indicating additional genetic changes accumulated during the evolution of this clade. Samples assigned to D.3.1.1 were characterized by the accumulation of additional amino acid substitutions in the HA1 subunit, including HA1: R113K, HA1: A139D, HA1: E283K, and HA1: K302E.

Within clade D.3.1, isolate 40,000,349 carried the HA1: D222N substitution, corresponding to the replacement of aspartic acid, D, by asparagine, N, at position 222. In clade D.3.1.1, isolate 40,000,443 carried the HA1: K169R substitution, suggesting additional local diversification within the HA segment.

The influenza A/SARS-CoV-2 co-infection isolate, 40,000,448, showed the characteristic mutation profile of influenza A(H1N1)pdm09 clade D.3.1.1, namely HA1: R113K, HA1: T120A, HA1: A139D, HA1: T216A, HA1: E283K, HA1: K302E, HA2: I45V, HA2: I133T, and HA2: V193A, with no additional amino acid substitutions detected.

**Figure 1:**
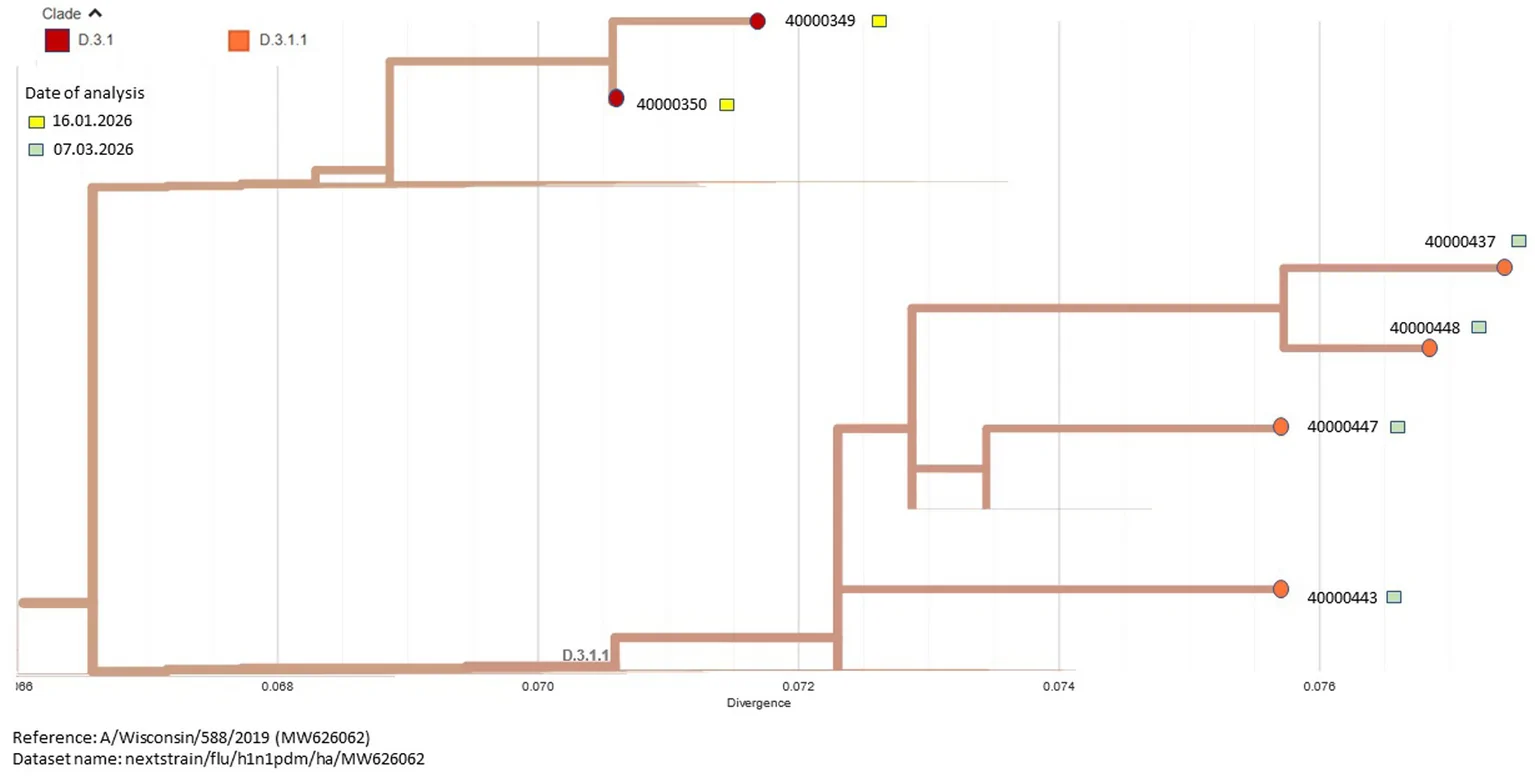
Phylogenetic placement of A(H1N1)pdm09 components in Nextclade, clades D.3.1 and D.3.1.1.

Phylogenetic analysis of the influenza A(H1N1)pdm09 sequences showed separation into two closely related genetic groups, D.3.1 and D.3.1.1, forming two distinct branches descending from clade D.3. The D.3.1 sequences clustered on the upper branch and were characterized by a stable mutation profile. Additional divergence within this branch was explained by the acquisition of the HA1: D222N amino acid substitution in isolate 40,000,349 (Figure 1). The D.3.1.1 sequences formed a separate branch, defined by the accumulation of four additional substitutions in the HA1 subunit. Isolate 40,000,443 clustered on a separate sub-branch because of the additional HA1: K169R substitution, which was not part of the core mutation profile of its assigned clade (Figure 1).

### 3.6 Characterization of A(H3N2

All 32 A(H3N2) components were assigned to clade K, subclade 2a.3a.1, corresponding to genetic lineage 3C.2a1b.2a.2a.3a.1. Compared with the reference sequence used in Nextclade, the A(H3N2) sequences showed 37–46 mutations, including 14–39 amino acid substitutions, 4–39 insertions, and 0–17 deletions. Common substitutions detected across the A(H3N2) dataset included HA1: K2N, HA1: N122D, HA1: T135K, HA1: S144N, HA1: N158D and HA2: S49N.

Visualization of the Moldovan A(H3N2) sequences on the Nextclade global reference tree showed that the sequences occupied three visually distinguishable groupings within clade K and differed in their amino acid substitution profiles. Sequences positioned in the upper part of the tree carried the HA1: R201I and HA1: A328T substitutions. Four sequences positioned in the middle part shared the HA1: F79I substitution. Sequences positioned in the lower part carried additional substitutions, including HA1: S95N, HA2: F138L, HA1: K82E, HA1: A212S, and HA1: S91R, which were not observed in the other Moldovan sequences included in this analysis. These visually distinguishable groupings are reported descriptively and should not be interpreted as evidence of separate introductions, distinct local transmission chains, or independent local evolution.

**Figure 2:**
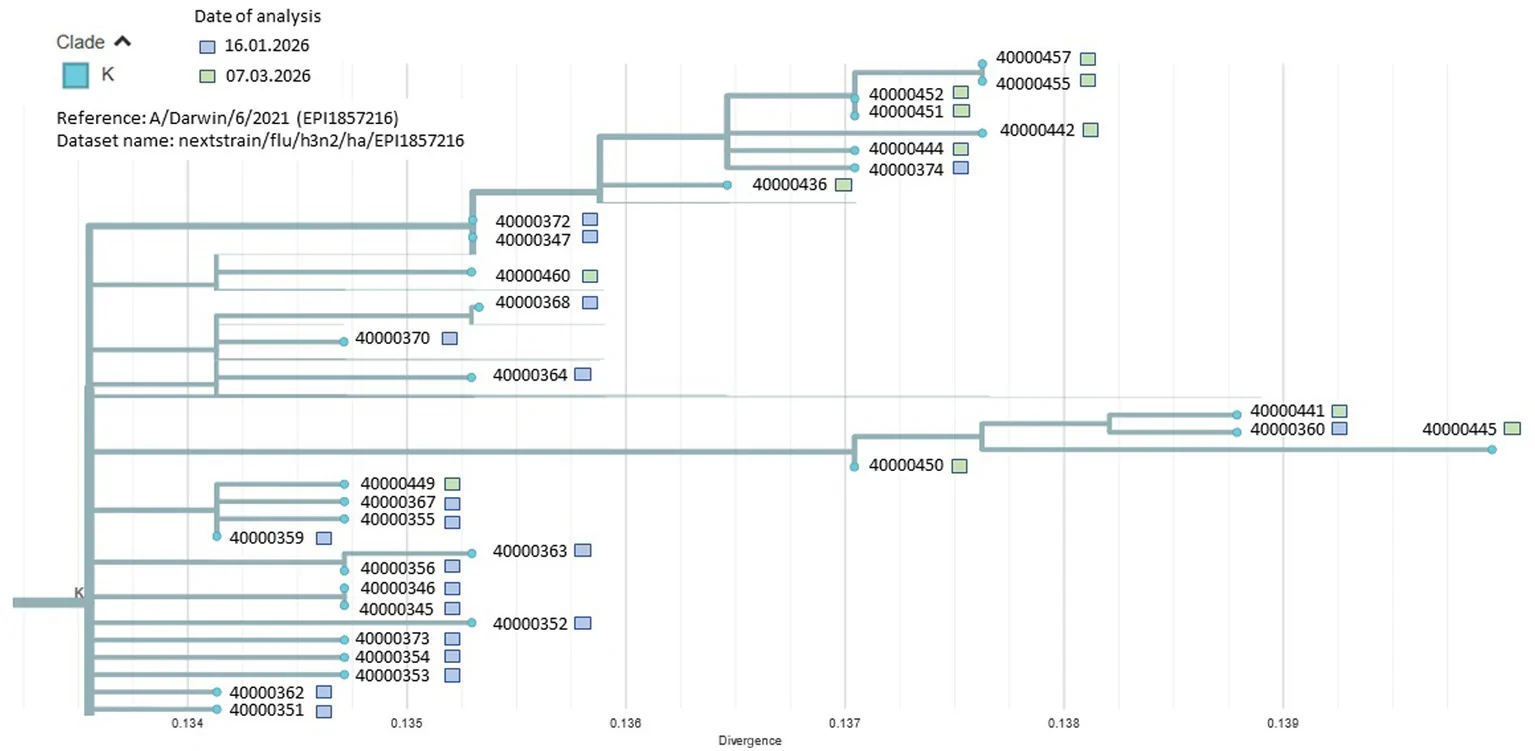
Phylogenetic placement of A(H3N2) components in Nextclade, Clade K.

These variations may reflect local micro-diversification or multiple introductions within an already dominant clade. However, in the absence of additional epidemiological and clinical data, they do not allow conclusions to be drawn regarding transmissibility or disease severity. All sequences clustered within clade K/subclade 2a.3a.1, with internal diversification into three groups (Figure 2).

### 3.7 Characterization of the influenza a/SARS-CoV-2 co-infection specimen and SARS-CoV-2 component

The mixed A(H1N1)pdm09/SARS-CoV-2 specimen, 40,000,448, was collected in Chișinău from a 10-year-old pediatric patient and was recorded under the COVID-19 diagnostic/surveillance category. Symptom onset was reported on 23 February 2026, and the specimen was collected on 25 February 2026. According to the available metadata, the patient was unvaccinated. The primary H1 Ct value was 18.65, whereas the repeated H1 Ct value was 25.91 and the SARS-CoV-2 Ct value was 26.47. Because these values were obtained using different molecular targets and the H1 Ct differed between the primary and repeated analyses, they cannot be directly compared as quantitative measures of viral load. The lower primary H1 Ct is compatible with a higher abundance of H1 target RNA in the initial assay, but this interpretation was not confirmed by the repeated H1 result.

**Figure 3:**
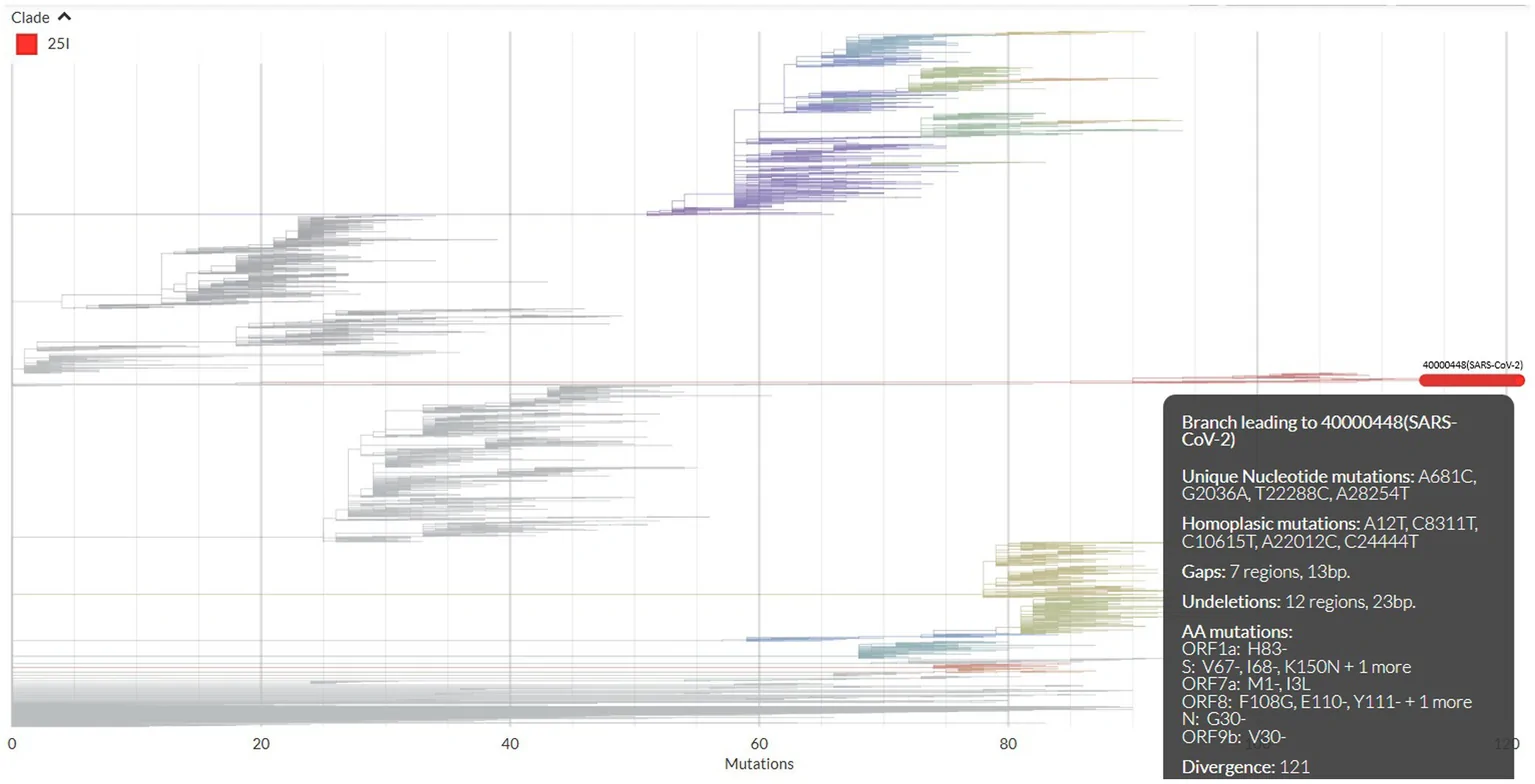
Nextclade phylogenetic placement of the SARS-CoV-2 component detected in the Influenza A/SARS-CoV-2 co-infection specimen.

The SARS-CoV-2 component was phylogenetically assigned to Nextclade/Nextstrain clade 25I and classified by Pango as sublineage SJ.1. This SARS-CoV-2 strain represents a genetic lineage distinct from JN.1 and its descendants, which predominated in the Republic of Moldova during 2024–2025. Nextclade analysis identified 165 mutations in the SARS-CoV-2 genome, including 23 amino acid substitutions, 18 insertions, and 87 deletions, as well as the private mutations in open reading frame 7a (ORF7a: I3L), open reading frame 8 (ORF8: F108G), and the spike protein (S: K150N). However, because the overall QC of the SARS-CoV-2 sequence was poor, these mutations should not be interpreted as robust findings, but rather as preliminary observations derived from the available consensus sequence. Sample 40,000,448 is shown in the SARS-CoV-2 reference tree; however, fine-scale interpretation of its mutational profile is limited by poor sequence quality (Figure 3).

## 4 Discussion

This study provides a descriptive HA-based genomic characterization of a laboratory-selected subset of influenza A-positive specimens collected through routine respiratory surveillance in the Republic of Moldova. It illustrates the application of multiplex RT-PCR screening, influenza-specific confirmatory testing, hybrid-capture sequencing, and HA-focused bioinformatic analysis, including genomic characterization of one mixed A(H1N1)pdm09/SARS-CoV-2 specimen previously identified by RT-PCR.

These findings complete the emerging national experience in the use of genomics for public health surveillance. Previous local studies on SARS-CoV-2 genetic diversity, wastewater-based genomic surveillance, and the application of metagenomic approaches to microorganisms of public health importance indicate the progressive development of NGS capacity in the Republic of Moldova (8, 9, 11, 14).

The predominance of clade K in the sequenced dataset is consistent with recent European data. ECDC reported an early increase in the circulation of this clade in the EU/EEA (20). A multicentre European vaccine-effectiveness study for the 2025/26 season showed that most sequenced viruses belonged to subclade K (21). Recent publications from 2026 have further described subclade K as a rapidly evolving antigenic variant of considerable public health relevance in Europe (22). The Moldovan data are aligned with this broader European context, although the selected sample size was too small to support definitive conclusions on the extent of population-level circulation.

The shared substitutions identified through genomic analysis of the A(H3N2) isolates, including HA1: K2N, HA1: T135K, HA1: S144N, HA1: N158D, and HA2: S49N, support their assignment to clade K. Submission of these sequences to GISAID or relevant national databases would provide additional value for regional epidemiological analyses and comparative genomic surveillance. The internal diversification into three distinct clusters may reflect multiple introductions, local evolution, or a combination of both processes.

For A(H1N1)pdm09, the identification of clades D.3.1 and D.3.1.1 within lineage 6B.1A.5a.2a.1 is consistent with the recent evolution of seasonal H1N1 viruses. Recent studies of A(H1N1)pdm09 have reported the accumulation of substitutions in the HA and NA segments, including within antigenically relevant regions, with potential implications for immune recognition (31). In the present analysis, the influenza A/SARS-CoV-2 co-infection specimen showed the characteristic mutational profile of clade D.3.1.1, with no additional amino acid substitutions in HA. This suggests that, at the hemagglutinin level, the A(H1N1)pdm09 component involved in the co-infection was not genetically distinct from the other D.3.1.1 isolates included in this study.

The present influenza analysis was restricted to the HA segment. The NA segment and the remaining influenza genome segments were not included in the downstream analysis; consequently, NA substitutions and markers potentially associated with antiviral resistance were not evaluated.

The D222N substitution was detected in one A(H1N1)pdm09 sequence. Substitutions at HA position 222, particularly D222G, have been discussed in relation to receptor-binding properties and severe disease (32). However, the clinical relevance of D222N remains uncertain. Given the absence of detailed clinical information and an appropriate comparison group, this finding is reported descriptively and should not be interpreted as evidence of disease severity in the corresponding patient. K169R and the other substitutions detected in individual isolates were likewise reported without attributing patient-level clinical significance.

The RT-PCR identification and subsequent genomic characterization of the mixed A(H1N1)pdm09/SARS-CoV-2 specimen illustrate the complementary roles of molecular diagnosis and targeted sequencing during periods of respiratory-virus co-circulation. Regional data support the value of integrated SARS-CoV-2/influenza surveillance, particularly when major respiratory viruses are circulating concurrently. In Bulgaria, RT-PCR-based studies identified SARS-CoV-2/influenza A(H3N2) co-infections during the 2021/2022 season, while subsequent analyses using multiplex polymerase chain reaction and NGS documented co-infections involving A(H1N1)pdm09 and A(H3N2), including sequencing of influenza A genomes from mixed specimens (33, 34). These findings are relevant to the Republic of Moldova because of geographical vicinity and similar needs for molecular surveillance. Comparable findings have also been reported in Italy. In Tuscany, among 940 SARS-CoV-2-positive samples tested during the 2021/2022 influenza season, 43 showed co-infection with influenza A, most of which were A(H3N2) (35). Italian clinical reports have described molecularly confirmed SARS-CoV-2/influenza A co-infections, including cases associated with acute respiratory distress syndrome or superinfection documented by detection of RNA from both pathogens (36, 37). In Spain, influenza A co-infection was also reported in patients with bilateral pneumonia, including during the early phase of the pandemic (38, 39). In this context, the identification of a mixed A(H1N1)pdm09/SARS-CoV-2 specimen in the present study supports the need to maintain multiplex testing and integrated sequencing for major respiratory viruses. However, a single co-infection case cannot be used to estimate population frequency or disease severity. Rather, it illustrates the complementary role of genomic sequencing following molecular detection.

The lower primary H1 Ct value compared with the SARS-CoV-2 Ct value is compatible with a higher relative abundance of influenza A target RNA in the initial assay. However, because the Ct values were obtained using different assays and molecular targets, they cannot be directly compared as quantitative measures of viral load. Moreover, the repeated H1 Ct value was close to the SARS-CoV-2 Ct value, further limiting interpretation of the difference between the initial results. The poorer quality of the SARS-CoV-2 consensus may have been influenced by lower target abundance, but other factors, including RNA degradation, hybrid-capture efficiency, competition between viral targets during library enrichment, and stochastic sequencing coverage, may also have contributed. Consequently, the SARS-CoV-2 consensus was retained only for broad clade-level interpretation, and mutation-level findings were not used for functional or epidemiological inference.

### 4.1 Limitations

This study has several important limitations.

The 38 specimens constituted a Ct-prioritized, laboratory-selected subset and were not statistically representative of all influenza A-positive specimens identified during the study period. Clinical and epidemiological metadata were incomplete: vaccination status was not reported for 7 of the 38 specimens, and the collection date was unavailable for 10 specimens. Beyond the AURTI /SARI case classification, detailed clinical information on disease severity was unavailable, precluding assessment of potential associations between the observed genomic characteristics and clinical presentation or severity. Furthermore, the data reflect a geographical imbalance due to the overrepresentation of Chișinău city, the short surveillance period, and a limited number of severe acute respiratory infection cases (SARI). These limitations prevent the estimation of the national prevalence of clades, the frequency of coinfections, and vaccine effectiveness.

Initial systematic diagnostic testing included influenza A, influenza B, and SARS-CoV-2 but did not include respiratory syncytial virus, parainfluenza viruses, adenoviruses, or other respiratory viruses. The presence and frequency of co-detections involving these pathogens could not be assessed.

From a bioinformatic point of view, downstream influenza analysis was restricted to the HA segment. The NA segment and the remaining influenza genome segments were not included in the final analysis; therefore, substitutions associated with antiviral resistance were not assessed. The phylogenetic framing was based on positioning in a reference tree instead of resorting to a dedicated phylogeographic analysis. At the same time, the SARS-CoV-2 sequence was marked by weaker QC, and the reported private mutations lacked subsequent confirmation by complementary methods.

The visually distinguishable positions of the Moldovan A(H3N2) sequences within the Nextclade reference tree should be interpreted cautiously. Because the study did not include comprehensive regional sampling, pairwise genomic-distance analysis, time-resolved phylogenetic reconstruction, or epidemiological linkage data, it was not possible to determine whether the observed patterns represented multiple introductions, local transmission, or independent evolutionary events.

### 4.2 Strengths

This study documents the development and application of genomic sequencing capabilities in respiratory virus surveillance in the Republic of Moldova. The laboratory workflow linked multiplex RT-PCR screening, influenza-specific confirmatory testing, hybrid-capture sequencing, HA-focused bioinformatic analysis, anonymized surveillance metadata, and international sequence-data sharing. Despite the fact that the sampling presents a certain territorial imbalance, the study includes samples from several geographical areas and provides a detailed clade-level characterization of both influenza A(H3N2) and A(H1N1)pdm09 viruses.

An important contribution is the inclusion of sequences of clade K of the A(H3N2) virus from Moldova, dating from a period when this subclade was epidemiologically relevant at the European level. This study also reports a molecularly detected coinfection with influenza A and SARS-CoV-2, addressing it in isolation without making unwarranted attempts to estimate the frequency of the phenomenon based on a single case. Also, interpretations regarding mutational profile were limited because of the lower quality of the consensus sequence for the SARS-CoV-2.

Another strength is the uploading the generate sequences to international public repositories, thereby increasing their value and facilitating comparative surveillance and epidemiological analyses at the regional level.

## 5 Conclusion

This study presents a descriptive genomic characterization of 38 laboratory-selected respiratory specimens positive for influenza A virus collected as part of routine epidemiological surveillance in the Republic of Moldova. All 32 A(H3N2) HA sequences were assigned to clade K (subclade 2a.3a.1), while six A(H1N1)pdm09 HA components were assigned to clusters D.3.1 or D.3.1.1. One case of A(H1N1)pdm09/SARS-CoV-2 coinfection was also identified.

The results demonstrate the feasibility of applying multiplex RT-PCR screening, influenza-specific confirmatory testing, hybrid-capture sequencing, and HA-focused bioinformatic analysis to laboratory-selected influenza A-positive specimen. Because of the insufficient quality of the SARS-CoV-2 consensus sequence, its interpretation was limited to clade assignment. The findings provide a descriptive snapshot of HA genetic diversity among the selected influenza A specimens and should not be interpreted as evidence of national clade predominance, geographical distribution, or co-detection frequency. The findings provide a descriptive snapshot of HA genetic diversity among the selected influenza A specimens and should not be interpreted as evidence of national clade predominance, geographical distribution, or co-detection frequency.

## Data Availability

The datasets presented in this study can be found in online repositories. The names of the repository/repositories and accession number(s) can be found at: https://gisaid.org, EPI_ISL_20325032; https://gisaid.org, EPI_ISL_20325033; https://gisaid.org, EPI_ISL_20388903; https://gisaid.org, EPI_ISL_20388961; https://gisaid.org, EPI_ISL_20388964; https://gisaid.org, EPI_ISL_20388976; https://gisaid.org, EPI_ISL_20324998; https://gisaid.org, EPI_ISL_20324999; https://gisaid.org, EPI_ISL_20325031; https://gisaid.org, EPI_ISL_20325035; https://gisaid.org, EPI_ISL_20325072; https://gisaid.org, EPI_ISL_20325074; https://gisaid.org, EPI_ISL_20325091; https://gisaid.org, EPI_ISL_20325127; https://gisaid.org, EPI_ISL_20325128; https://gisaid.org, EPI_ISL_20325129; https://gisaid.org, EPI_ISL_20325131; https://gisaid.org, EPI_ISL_20325132; https://gisaid.org, EPI_ISL_20325134; https://gisaid.org, EPI_ISL_20325135; https://gisaid.org, EPI_ISL_20325137; https://gisaid.org, EPI_ISL_20325167; https://gisaid.org, EPI_ISL_20325168; https://gisaid.org, EPI_ISL_20325169; https://gisaid.org, EPI_ISL_20325170; https://gisaid.org, EPI_ISL_20325173; https://gisaid.org, EPI_ISL_20388810; https://gisaid.org, EPI_ISL_20388904; https://gisaid.org, EPI_ISL_20388905; https://gisaid.org, EPI_ISL_20388962; https://gisaid.org, EPI_ISL_20388963; https://gisaid.org, EPI_ISL_20388988; https://gisaid.org, EPI_ISL_20389020; https://gisaid.org, EPI_ISL_20389078; https://gisaid.org, EPI_ISL_20389118; https://gisaid.org, EPI_ISL_20389147; https://gisaid.org, EPI_ISL_20389148; https://gisaid.org, EPI_ISL_20389149; and https://gisaid.org, EPI_ISL_20388977.

## Glossary

ANI: Average nucleotide identity
AURTI: Acute upper respiratory tract infection
COVID-19: Coronavirus disease 2019
Ct: Cycle threshold
DNA: Deoxyribonucleic acid
DRAGEN: Dynamic Read Analysis for GENomics
ECDC: European Centre for Disease Prevention and Control
EU/EEA: European Union/European Economic Area
FASTA: Text-based format for representing nucleotide or amino acid sequences
FASTQ: Text-based format for representing nucleotide sequences and corresponding quality scores
GISAID: Global Initiative on Sharing All Influenza Data
GISRS: Global Influenza Surveillance and Response System
HA: Hemagglutinin
HA1: Hemagglutinin subunit 1
HA2: Hemagglutinin subunit 2
ILI: Influenza-like illness
NA: Neuraminidase
NGS: Next-generation sequencing
ORF: Open reading frame
QC: Quality control
RNA: Ribonucleic acid
RSV: Respiratory syncytial virus
RT-PCR: Reverse transcription polymerase chain reaction
SARI: Severe acute respiratory infection
SARS-CoV-2: Severe acute respiratory syndrome coronavirus 2
SNP: Single-nucleotide polymorphism
WHO: World Health Organization

## References

[1] World Health Organization. Influenza (seasonal). Geneva: WHO (2025).

[2] World Health Organization. Implementing the Integrated Sentinel Surveillance of influenza and other Respiratory Viruses of Epidemic and Pandemic Potential by the Global Influenza Surveillance and Response System: Standards and Operational Guidance. Geneva: WHO (2024).

[3] World Health Organization. Global Genomic Surveillance Strategy for Pathogens with Pandemic and Epidemic Potential, 2022–2032. Geneva: WHO (2022). 10.2471/BLT.22.288220 PMC8958828 35386562

[4] European Centre for Disease Prevention and Control. Reporting Protocol for Integrated Respiratory Virus Surveillance, Version 1.1. Stockholm: ECDC (2026).

[5] Druc A. Evolution of influenza and acute respiratory infections in the Republic of Moldova during the 2014/15 to 2022/23 epidemic seasons. One Health Risk Manag. (2024) 5:12–20. doi: 10.38045/ohrm.2024.3.02

[6] Colac S. Genetic significance and monitoring of circulating variants of the SARS-CoV-2 virus in the Republic of Moldova. One Health Risk Manag. (2023) 2:85. Available online at: https://ibn.idsi.md/ro/vizualizare_articol/192303/cerif

[7] Colac S. Variabilitatea genetică a virusului SARS-CoV-2, varianta omicron circulante pe teritoriul Republicii Moldova / genetic variability of the omicron variant of the SARS-CoV-2 virus circulating in the territory of the Republic of Moldova. Revista de Științe ale Sănătății din Moldova. (2023) 10:152. Available online at: https://ibn.idsi.md/ro/vizualizare_articol/232233

[8] Colac S Ulinici M Burduniuc O. Analiza diversităţii genetice a virusului SARS-CoV-2: revista literaturii. One Health Risk Manag. (2024):136. Available online at: https://ibn.idsi.md/en/vizualizare_articol/196573/datacite

[9] Colac S Ulinici M Burduniuc O. Genetic diversity analysis of the SARS-CoV-2 virus: a literature review. One Health Risk Manag. (2025) 6:16–28. doi: 10.38045/ohrm.2025.1.02

[10] Colac S Sitnic V Burduniuc O. Genomic monitoring of SARS-CoV-2 variants in the Republic of Moldova. Arta Med. (2025) 94:11–6. doi: 10.5281/zenodo.15879875

[11] Colac S. "Analiza secvențierii genomului SARS-CoV-2: evoluția și impactul noilor linii Omicron în Republica Moldova / SARS-CoV-2 genome sequence analysis: evolution and impact of new Omicron lineages in the Republic of Moldova". In: Condraticova L, editor. Patrimoniul cultural de ieri – implicaţii în dezvoltarea societăţii durabile de mâine. Ediția XI; 11–12 februarie 2025. Iași-Chișinău-Lviv: Authentication and Conservation of Cultural Heritage, Research and Technique (2025). p. 280.

[12] Colac S. "Monitoring the spread of SARS-CoV-2 via wastewater genome sequencing". In: Cebanu S, editor. Abordarea O singură sănătate pentru securitatea sănătăţii globale; 20–21 noiembrie 2025; Chişinău. Chişinău: Tipogr. Print Caro (2025). p. 18.

[13] Țapu L Lupu M Colac S Burduniuc O Bucov V. Supravegherea rezistenței la antimicrobiene bazată pe tehnologia metagenomică. Arta Med. (2026) 98:147. Available online at: https://ibn.idsi.md/vizualizare_articol/244961

[14] Colac S Burlac V Sîtnic V. "Determinanți genetici și ai virulenței în genomul microorganismelor cu impact major asupra sănătății publice". In: Cebanu S, editor. Abordarea O singură sănătate pentru securitatea sănătăţii globale; 20–21 noiembrie 2025; Chişinău. Chişinău: Tipogr. Print Caro (2025). p. 15.

[15] Colac S Burduniuc O Apostol M. "Monitorizarea infecției COVID-19 prin secvențierea întregului genom și analiza filogenetică a izolatelor SARS-CoV-2". In: Cercetarea în biomedicină și sănătate: calitate, excelență și performanță. Culegere de postere electronice; 19–21 octombrie 2022; Chişinău. Chişinău: Nicolae Testemitanu State University of Medicine and Pharmacy of the Republic of Moldova (2022). p. 166.

[16] Colac S Burduniuc O Apostol M. Monitoring COVID-19 infection through whole-genome sequencing and phylogenetic analysis of SARS-CoV-2 isolates. Mold J Health Sci. (2022) 29:136. Available online at: https://ibn.idsi.md/vizualizare_articol/168334

[17] Shu Y McCauley J. GISAID: global initiative on sharing all influenza data – from vision to reality. Euro Surveill. (2017) 22:30494. doi: 10.2807/1560-7917.ES.2017.22.13.30494, 28382917 PMC5388101

[18] Aksamentov I Roemer C Hodcroft EB Neher RA. Nextclade: clade assignment, mutation calling and quality control for viral genomes. J Open Source Softw. (2021) 6:3773. doi: 10.21105/joss.03773

[19] Rambaut A Holmes EC O’Toole Á Hill V McCrone JT Ruis C . A dynamic nomenclature proposal for SARS-CoV-2 lineages to assist genomic epidemiology. Nat Microbiol. (2020) 5:1403–7. doi: 10.1038/s41564-020-0770-5, 32669681 PMC7610519

[20] European Centre for Disease Prevention and Control. Threat Assessment Brief: Assessing the risk of influenza for the EU/EEA in the context of Increasing Circulation of A(H3N2) Subclade K. Stockholm: ECDC (2025).

[21] Lucaccioni H Marques DFP Kirsebom F Emborg H-D Hamilton M Whitaker H . Influenza vaccine effectiveness from nine studies during drifted a(H3N2) subclade K predominance, Europe, September 2025 to January 2026. Euro Surveill. (2026) 31:2600109. doi: 10.2807/1560-7917.ES.2026.31.7.2600109, 41716088 PMC12924001

[22] Scarvaglieri I De Francesco MA Alberti M Cesanelli F Salvi M Tiecco G . Influenza a(H3N2) subclade K: epidemiology, molecular evolution and vaccine effectiveness in Europe. Pathogens. (2026) 15:474. doi: 10.3390/pathogens15050474, 42198601 PMC13209883

[23] World Health Organization. Recommended Composition of influenza Virus Vaccines for use in the 2026–2027 Northern Hemisphere influenza Season. Geneva: WHO (2026).

[24] Wu X Cai Y Huang X Yu X Zhao L Wang F . Co-infection with SARS-CoV-2 and influenza a virus in patient with pneumonia, China. Emerg Infect Dis. (2020) 26:1324–6. doi: 10.3201/eid2606.200299, 32160148 PMC7258479

[25] Stowe J Tessier E Zhao H Guy R Muller-Pebody B Zambon M . Interactions between SARS-CoV-2 and influenza, and the impact of coinfection on disease severity: a test-negative design. Int J Epidemiol. (2021) 50:1124–33. doi: 10.1093/ije/dyab081, 33942104 PMC8135706

[26] Swets MC Russell CD Harrison EM Docherty AB Lone N Girvan M . SARS-CoV-2 co-infection with influenza viruses, respiratory syncytial virus, or adenoviruses. Lancet. (2022) 399:1463–4. doi: 10.1016/S0140-6736(22)00383-X, 35344735 PMC8956294

[27] Dadashi M Khaleghnejad S Abedi Elkhichi P Goudarzi M Goudarzi H Taghavi A . COVID-19 and influenza co-infection: a systematic review and meta-analysis. Front Med. (2021) 8:681469. doi: 10.3389/fmed.2021.681469, 34249971 PMC8267808

[28] Zheng J Chen F Wu K Wang J Li F Huang S . Clinical and virological impact of single and dual infections with influenza a(H1N1) and SARS-CoV-2 in adult inpatients. PLoS Negl Trop Dis. (2021) 15:e0009997. doi: 10.1371/journal.pntd.0009997, 34843492 PMC8659415

[29] Golpour M Jalali H Alizadeh-Navaei R Rezaei Talarposhti M Mousavi T Nadi Ghara AA. Co-infection of SARS-CoV-2 and influenza a/B among patients with COVID-19: a systematic review and meta-analysis. BMC Infect Dis. (2025) 25:145. doi: 10.1186/s12879-025-10521-5, 39891054 PMC11783914

[30] Ministry of Health of the Republic of Moldova. Ordinul nr. 897 din 8 octombrie 2025 cu privire la supravegherea epidemiologică a infecțiilor respiratorii virale acute [Order No. 897 of 8 October 2025 on the epidemiological surveillance of acute viral respiratory infections]. Chișinău: Ministry of Health of the Republic of Moldova (2025).

[31] Wang N Lu W Yan L Liu M Che F Wang Y . Epidemiological and genetic characterization of the influenza a(H1N1) virus in Hangzhou City in 2023. Front Public Health. (2024) 12:1464435. doi: 10.3389/fpubh.2024.1464435, 39635219 PMC11614803

[32] Kolosova NP Boldyrev ND Svyatchenko SV Danilenko AV Goncharova NI Shadrinova KN . An investigation of severe influenza cases in Russia during the 2022–2023 epidemic season and an analysis of HA-D222G/N polymorphism in newly emerged and dominant clade 6B.1A.5a.2a A(H1N1)pdm09 viruses. Pathogens. (2024) 13:1. doi: 10.3390/pathogens13010001, 38276147 PMC10819184

[33] Trifonova I Korsun N Madzharova I Alexiev I Ivanov I Levterova V . Epidemiological and genetic characteristics of respiratory viral coinfections with different variants of severe acute respiratory syndrome coronavirus 2 (SARS-CoV-2). Viruses. (2024) 16:958. doi: 10.3390/v16060958, 38932250 PMC11209099

[34] Trifonova I Christova I Madzharova I Angelova S Voleva S Yordanova R . Clinical significance and role of coinfections with respiratory pathogens among individuals with confirmed severe acute respiratory syndrome coronavirus-2 infection. Front Public Health. (2022) 10:959319. doi: 10.3389/fpubh.2022.959319, 36117597 PMC9479447

[35] Milano G Marchi S Vicenti I Biba C Fiaschi L Trombetta CM . SARS-CoV-2 and influenza virus coinfections in the Tuscan population during the 2021/2022 influenza season. J Prev Med Hyg. (2024) 65:E11–6. doi: 10.15167/2421-4248/jpmh2024.65.1.3179, 38706768 PMC11066830

[36] Arcangeletti MC De Conto F Montecchini S Buttrini M Maccari C Chezzi C . A rare case of SARS-CoV-2 and influenza a virus super-infection. Diagn Microbiol Infect Dis. (2022) 104:115743. doi: 10.1016/j.diagmicrobio.2022.115743, 35834915 PMC9212775

[37] D’Abramo A Lepore L Palazzolo C Barreca F Liuzzi G Lalle E . Acute respiratory distress syndrome due to SARS-CoV-2 and influenza a co-infection in an Italian patient: Mini-review of the literature. Int J Infect Dis. (2020) 97:236–9. doi: 10.1016/j.ijid.2020.06.056, 32565366 PMC7301795

[38] Cuadrado-Payán E Montagud-Marrahi E Torres-Elorza M Bodro M Blasco M Poch E . SARS-CoV-2 and influenza virus co-infection. Lancet. (2020) 395:e84. doi: 10.1016/S0140-6736(20)31052-7, 32423586 PMC7200126

[39] Lozano-Parras MA Amann-Arévalo M Ciller-Martínez M Culebras-López E. COVID-19 and influenza a coinfection: a matter of principle. Enferm Infecc Microbiol Clin. (2021) 39:214–5. doi: 10.1016/j.eimce.2020.06.009, 38620657 PMC7365066

